# Advanced glycation end products (AGEs) promote melanogenesis through receptor for AGEs

**DOI:** 10.1038/srep27848

**Published:** 2016-06-13

**Authors:** Eun Jung Lee, Ji Young Kim, Sang Ho Oh

**Affiliations:** 1 Department of Dermatology and Cutaneous Biology Research Institute, Severance Hospital, Yonsei University College of Medicine, Seoul, Korea

## Abstract

Accumulation of advanced glycation end products (AGEs) is linked with development or aggravation of many degenerative processes or disorders, including aging and atherosclerosis. AGEs production in skin cells is known to promote stiffness and loss of elasticity through their buildup in connective tissue. However, the impact of AGEs has yet to be fully explored in melanocytes. In this study, we confirmed the existence of receptor for AGE (RAGE) in melanocytes in western blot and immunofluorescence along with increased melanin production in *ex vivo* skin organ culture and *in vitro* melanocyte culture following AGEs treatment. Cyclic AMP response element-binding protein (CREB) and extracellular signal-regulated kinases (ERK) 1/2 are considered as key regulatory proteins in AGEs-induced melanogenesis. In addition, blockage experiment using anti-RAGE blocking antibody has indicated that RAGE plays a pivotal role in AGE-mediated melanogenesis. Therefore, it is apparent that AGEs, known markers of aging, promote melanogenesis via RAGE. In addition, AGEs could be implicated in pigmentation associated with photoaging according to the results of increased secretion of AGEs from keratinocytes following UV irradiation. AGE-mediated melanogenesis may thus hold promise as a novel mean of altering skin pigmentation.

Advanced glycation end products (AGEs) are generated via the Maillard reaction (i.e., nonenzymatic glycation/oxidation of proteins, lipids, and nucleic acids, which are covalently bonded to reducing sugars)^1^. Various structures of AGEs such as Nε-(carboxymethyl)-lysine (CML), pyrraline, pentosidine, or other crossslines^2^ are known to be associated with degenerative process or disorders, including aging^3^, diabetes, atherosclerosis^4^, Alzheimer’s disease^5^, and renal failure^6^. AGEs also implicated in skin aging, accumulate a result of UV irradiation^7^ in both senescent and photoaged skin^8^,^9^.

A variety of cutaneous cells, such as fibroblasts and keratinocytes^10^,^11^, produce AGEs, which are common affiliates of fibronectin, laminin, collagen, elastin^8^,^12^, and epidermis^13^. Accumulated AGEs in collagen and elastin of connective tissue lead to stiffening and loss of elasticity^14^. Furthermore, previous literatures have reported that UV-induced intracellular buildup of AGEs generates reactive oxygen species (ROS) damaging dermal proteins and triggering inflammatory signaling response. All of these factors attest to the putative influence of AGEs on the photoaging of skin such as wrinkling^15^,^16^.

UV irradiation, in addition to its role associated with wrinkling, can also clearly intensify skin pigmentation. Senile pigmentation (i.e., aged spots or solar lentigo) is another prominent manifestation of chronic actinic damage. Considering the already known association of AGEs with skin aging, a correlation between AGEs and UV-induced skin pigmentation also seems feasible. Previous studies have confirmed deposition of AGEs in skin tissues through immunohistochemical staining and two-dimensional polyacrylamide gel electrophoresis^3^,^17^. However, it is still uncertain how AGEs exert effects in the course of melanogenesis.

The receptor for advanced glycation end products (RAGE) is a multiligand member of the immunoglobulin superfamily of cell surface receptors that is expressed in various skin cells including fibroblasts, dendritic cells, and keratinocytes^7^. Upon ligand binding, increases in S100/calgranulins, amphoterin, and high mobility group box 1 (HMGB-1) generate ROS, and proinflammatory upregulation ensues^18^,^19^. Earlier studies have focused on blockade of RAGE, using anti-RAGE antibody or soluble RAGE (sRAGE) to reduce inflammation^20^,^21^,^22^ and demonstrated that RAGE signaling is involved in fibrosis and growth factor secretion^21^,^23^ and in matrix metalloproteinase-9 (MMP-9) activation in keratinocytes^24^. Nevertheless, the impact of AGEs and RAGE binding is still unclear in melanogenesis signaling.

In this study, we aimed to investigate the role of AGEs and RAGE in melanin production and examine related signaling mechanisms. Our findings provide evidence that AGEs promote melanogenesis through RAGE activation in melanocytes.

## Results

### RAGE expression in skin cells

**Figure 1:**
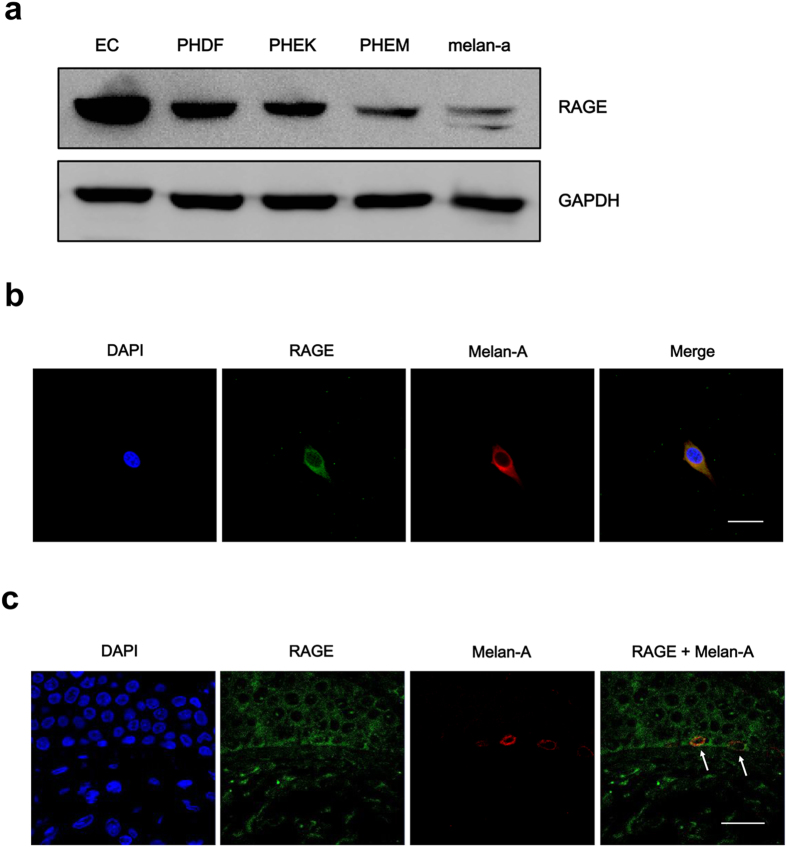
RAGE expression in skin cells. (**a**) RAGE protein expression was confirmed by western blot analysis. RAGE is expressed in skin cells, PHDF, PHEK, PHEM, and melan-a cell line. Human endothelial cells (EC) were used as positive control. (**b**) Immunofluorescence staining of RAGE in PHEM and (**c**) human skin tissue was performed. Melan-A (red) and RAGE (green) immunostained melanocytes were visualized in the basal layer of epidermis. Arrows indicate RAGE-expressing melanocytes. Scale bar represents 20 μm.

Before performing experiments on the effect of AGEs on melanogenesis, we investigated whether melanocytes express RAGE, the known receptor for AGEs. We tested the expression of RAGE in primary human dermal fibroblasts (PHDFs) and primary human epidermal keratinocytes (PHEKs) together with primary human epidermal melanocytes (PHEMs) using lysates of human endothelial cells (EC) as a positive control. Interestingly, PHEMs expressed RAGE as other primary human skin cells and their expressions were consistent with previous reports which showed the presence of RAGE in PHDFs and PHEKs^7^. In addition, since we have used mouse melanocyte cell line, melan-a, we checked RAGE expression on melan-a cell as well (Fig. 1a). For the confirmation of RAGE expression in melanocytes, we checked RAGE expression in PHEMs and skin tissues using melan-A antibody, a melanocyte marker via double immunofluorescence staining. We were able to observe melanocytes which stained with melan-A also expressed RAGE *in vitro* cell (Fig. 1b) and *in vivo* tissue (Fig. 1c). From these results, we found that RAGE expression is indeed present in melanocytes.

### AGEs promote epidermal pigmentation in organ-cultured human skin

**Figure 2:**
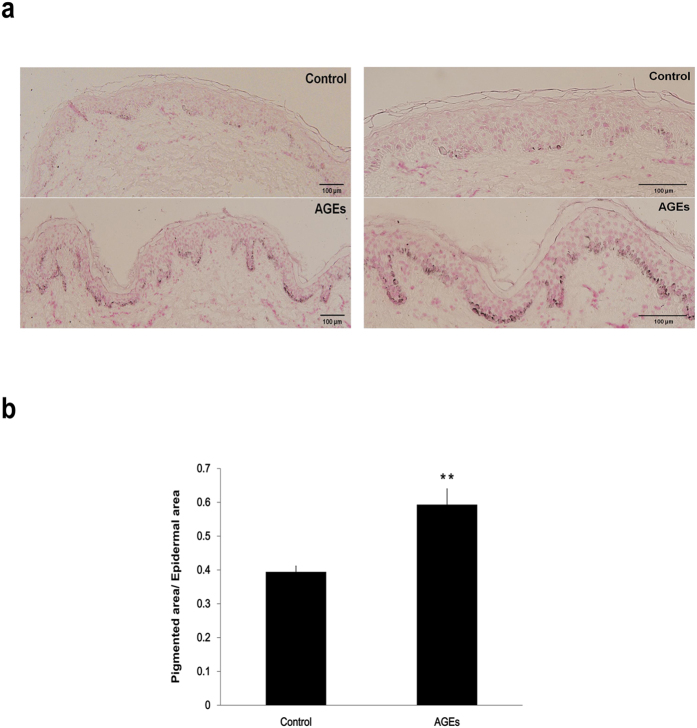
AGEs promote epidermal pigmentation in organ-cultured human skin. (**a**) Human skin cultures exposed to AGEs (200 μg/ml) twice (Day 1 and 3) in a 5-day period were stained for melanin (Fontana-Masson kit) as described by the manufacturer. Pigmentation increased in test samples compared with controls. Right column images are enlarged version of left panel. (**b**) Ratios of pigmented epidermal areas to total epidermal areas differed significantly in AGE-exposed and control skin sections, as show in bar graph. Scale bar represents 100 μm. **p < 0.01. All values expressed as mean ± SD of three independent experiments.

AGE-mediated melanogenesis was explored in *ex vivo* experiments using human skin. AGEs (200 μg/ml) were delivered twice (day 1 and 3) over a period of 5 days to an organ culture system which utilized remnants of breast skin from plastic surgery. As a result, increased melanin content was demonstrated (via Fontana-Masson staining) in skin cultures 5 days after AGE exposure (Fig. 2a). In AGE-exposed human skin (vs. non-exposed controls), the ratio of pigmented epidermal area to total epidermal areas was significantly greater (0.394 ± 0.017 versus 0.593 ± 0.047, **p < 0.01) (Fig. 2b). However, there was no difference in the number of epidermal melanocytes between both control and AGEs-delivered tissues (Fig. S1). Taken together, these results indicate that AGEs has stimulatory effect on pigmentation without increasing number of epidermal melanocytes.

### AGEs induce melanin production and increase expression of melanogenesis-associated molecules in melan-a cells

**Figure 3:**
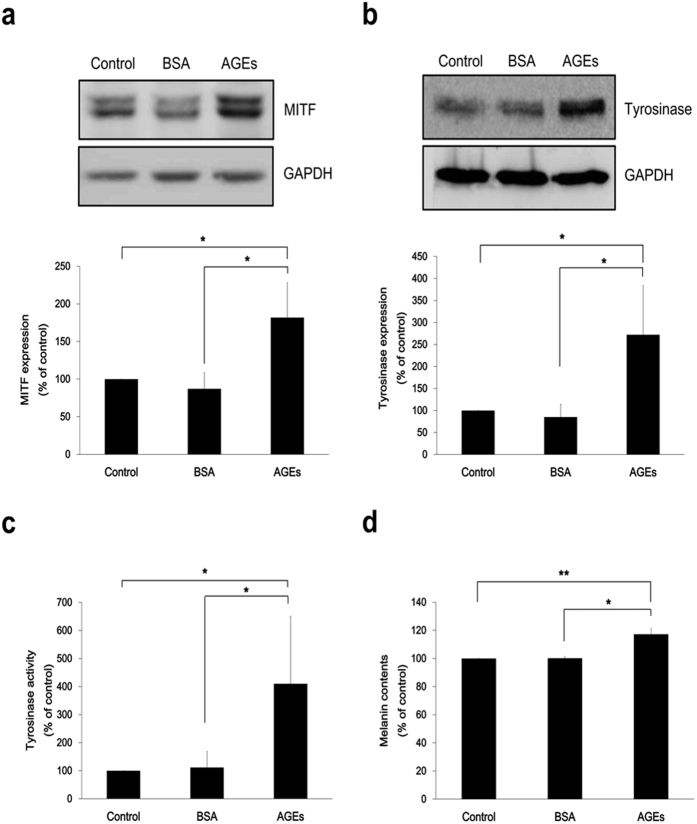
AGEs induce melanin production and increase expression of melanogenesis-associated molecules in melan-a cells. Media of melan-a cells were changed to serum free media and AGEs (200 μg/ml) were treated. (**a**) MITF levels were assayed after 4 h AGEs exposure. (**b**) Tyrosinase expression and (**c**) its activity were confirmed at Day 3 by western blot analysis and zymography, respectively. (**d**) Melanin content exposed to AGEs for 4 days was measured. *p < 0.05; **p < 0.01. All data expressed as mean ± SD of three independent experiments.

The melanogenic effect of AGEs was assessed in melan-a cells after first evaluating the impact of AGEs on cell viability. No cytotoxicity resulted from preliminary AGEs in bovine serum albumin (AGE-BSA), exposure for 4 days (Fig. S2) and AGEs treatment did not induce direct effect on melanocytes based on the results of ROS, cytokine (IL-6, TNF-α) and p38 response (Fig. S3). As cells were exposed to AGEs (200 μg/ml), expression levels of microphthalmia-associated transcription factor (MITF) and tyrosinase, the melanogenesis-associated molecules, were increased (Fig. 3a,b). In addition, tyrosinase enzymatic activity rose significantly upon incubation with AGEs (Fig. 3c), along with a significant increase in melanin content (~17%) compared to control cells (Fig. 3d, *p < 0.05; **p < 0.01). To see the effect of long incubation of AGEs on melanogenesis, we treated AGEs on melan-a cells for 2 weeks. Two week-incubation of AGEs augmented more melanin contents (~23.9%) in comparison to 4 day-incubation (Fig. S4).

### AGEs induce melanin production and increase expression of melanogenesis-associated molecules in PHEMs

**Figure 4:**
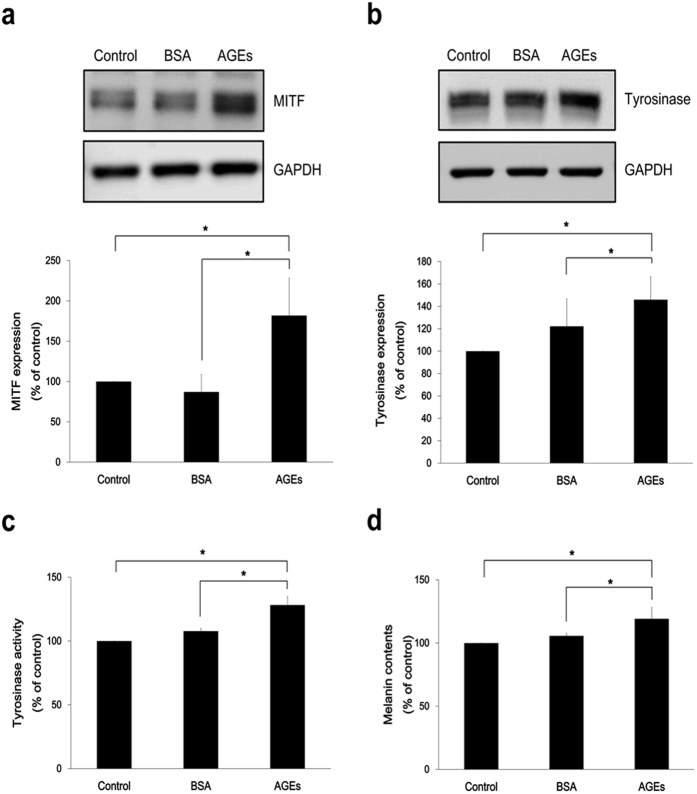
AGEs induce melanin production and increase expression of melanogenesis-associated molecules in primary human epidermal melanocytes. AGEs (200 μg/ml) were treated to PHEMs in serum free media. (**a**) After 4 h incubation, MITF levels were confirmed. (**b**) Tyrosinase expression and (**c**) activity were assayed at Day 2. (**d**) At Day 3, melanin content exposed to AGEs was measured. *p < 0.05. All data expressed as the mean ± SD of three independent experiments.

Melanogenesis signaling in PHEMs was also investigated. Treatment of AGEs on PHEMs resulted in increased protein levels of MITF (Fig. 4a) and tyrosinase (Fig. 4b), compared with control samples. Moreover, AGEs exposure in PHEMs promoted the increase of tyrosinase enzymatic activity (Fig. 4c) and melanin content (~24.4%) (Fig. 4d). The effect of longer incubation of AGEs on melanogenesis was observed in PHEMs as well. Longer incubation with AGEs (2 weeks) augmented melanin production up to ~28.5% (Fig. S4). Moreover, the increase in melanin production through AGEs was also observed in PHEMs from non-genital skin. (Fig. S5). These results confirm that melanogenesis singaling is activated by AGEs treatment in PHEMs along with melan-a cells.

### AGEs upregulate RAGE expression and its downstream signaling

**Figure 5:**
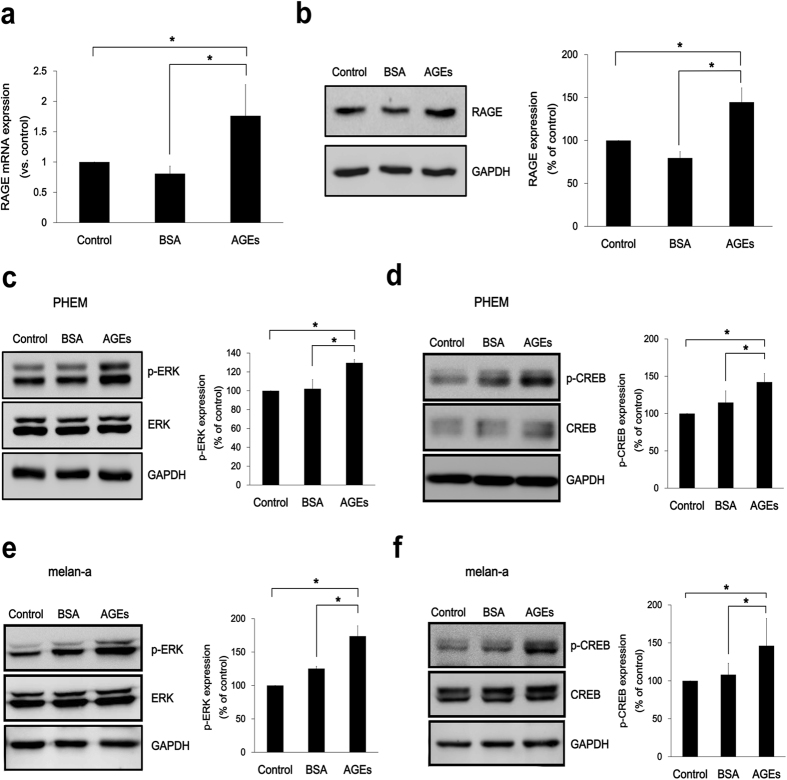
AGEs upregulate RAGE expression and its downstream signaling. (**a**) PHEMs were treated with AGEs (200 μg/ml) for 2 h in serum free media and RAGE mRNA expression was determined by realtime PCR. (**b**) RAGE protein levels were assayed after 4 h AGEs incubation. In PHEMs, (**c**) p-ERK1/2 and (**d**) p-CREB protein levels were determined after exposure to AGEs (200 μg/ml) in serum free media for 15 min and 30 min, respectively. In melan-a cells, protein levels of (**e**) p-ERK1/2 and (**f**) p-CREB were also measured. *p < 0.05. All data expressed as the mean ± SD of three independent experiments.

The level of RAGE in cells treated with AGEs was investigated. The upregulation of RAGE mRNA (Fig. 5a) and protein expression (Fig. 5b) was observed in cells with AGEs exposure. Downstream targets of RAGE signaling, ERK and CREB activation were evaluated to determine their relationship in AGE-mediated melanogenesis. Relative to controls, protein levels of p-ERK (Fig. 5c) and p-CREB (Fig. 5d) (phosphorylated states) were significantly increased by AGEs exposure in PHEMs. ERK and CREB phosphorylation was also detected in AGEs-treated melan-a cell line (Fig. 5e,f) Taken together, these results demonstrate that AGEs stimulate RAGE pathway by increasing RAGE expression and phosporylating ERK and CREB.

### Functional significance of RAGE in AGE-mediated melanogenesis

**Figure 6:**
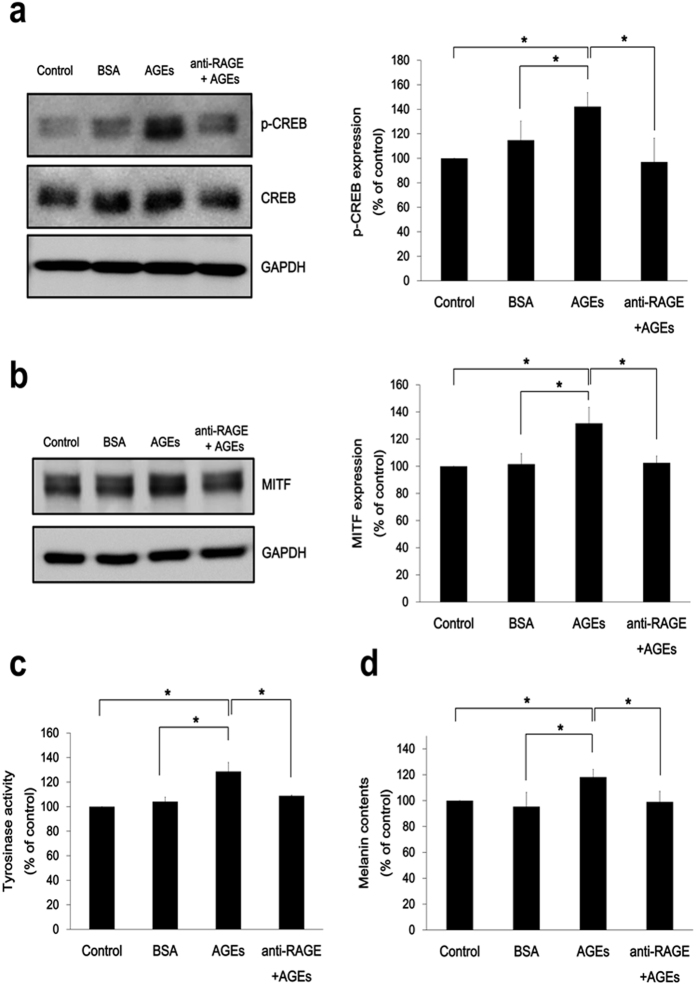
Functional significance of RAGE in AGE-mediated melanogenesis. To block AGE-RAGE interaction, PHEMs exposed (1 h) to anti-RAGE antibody (20 μg/ml) were incubated with AGEs (200 μg/ml) in serum free media. After 30 min, 4 h, 48 h, and 72 h, (**a**) p-CREB, (**b**) MITF expression, (**c**) tyrosinase activity, and (**d**) melanin content, respectively were assessed. *p < 0.05. All data expressed as the mean ± SD of three independent experiments.

We subsequently investigated whether the receptor for AGE (RAGE) participates in AGE-induced melanogenesis. To investigate the role of RAGE in AGE-mediated melanogenesis, cells that had been preincubated (1 h) in RAGE blocking antibody (R&D Systems)^25^ were tested for p-CREB and MITF expression after incubation with AGEs (200 μg/ml). The expected rise in expression of p-CREB and MITF (due to AGEs) was abrogated by prior RAGE blockade (Fig. 6a,b). Likewise, tyrosinase activity (Fig. 6c) and melanin synthesis (Fig. 6d) did not increase. These findings suggest that AGE-mediated melanogenesis relies on RAGE signaling.

## Discussion

Previous studies of AGEs have largely focused on related systemic processes or diseases, such as aging and diabetes^14^,^26^,^27^,^28^,^29^. AGEs have been found to accumulate in the blood vessels of diabetic patients, leading to excessive ROS production and quenching of nitric oxide activity in vessels^14^,^30^,^31^. In skin components, such as fibronectin, laminin, elastin, and collagen, accumulation of AGEs take the form of Nε-(carboxymethyl)-lysine (CML), pyrraline, pentosidine, or other molecules^2^ as consequences of aging and actinic damage. AGEs accumulation is fundamental in skin aging, as it leads to oxidative stress and inflammation^16^,^17^,^32^. High levels of AGEs likewise may impair dermal regeneration, reducing collagen solubility due to intermolecular crosslinking and matrix metalloproteinase resistance^7^,^33^,^34^. However, aside from the known contribution of AGEs to the wrinkling of aging skin, the interrelationship of AGEs and melanocytes has not been adequately studied.

Skin pigmentation is a frontline barrier to damage by internal and external stressors such as variable hormones and UV exposure^35^, and in conjunction with wrinkles, it is considered as a hallmark of photoaging. Since repeated UV exposure is a culprit of pigmentary changes and skin cancers^36^,^37^, mechanisms of UV damage and protective strategies have already been extensively researched^38^. UV irradiation induces oxidative stress, boosts proinflammatory cytokines and generates harmful AGEs in skin^17^,^39^. In addition, as skin is exposed to UV, melanocortin-1 receptor (MC1R) and its ligand, α-melanocyte-stimulating hormone (α-MSH) and adrenocorticotropic hormone (ACTH), are particularly responsive to stresses. The signal pathway through MC1R is critical in driving melanin synthesis^40^ by increasing intracellular cAMP levels^41^ and activating protein kinase A^42^. In our study, we investigated the contribution of AGEs-RAGE to pigmentation as an alternate mechanism of pigmentation signaling other than MC1R-mediated pathway and further sought to oberve the implication of AGEs-RAGE pathway in UV-induced pigmentation. And through our experiments, we were able to identify the expression of RAGE in melanocytes and the role of AGEs in melanogenesis. In order to support the possibility of the AGEs’ role in UV-induced pigmentation, we checked AGEs secretion and production from supernatant and lysates of keratinocytes after UV irradiation. Consequently, increased secretion and production of AGEs were observed from keratinocytes flollowing UV irradiation (Fig. S6).

In this study, a commercially available mixture of Nε-(carboxymethyl)-lysine, pentosidine, and others^43^, was used for seeing the effects of AGEs on melanogenesis in *ex vivo* skin organ culture and *in vitro* melanocyte culture. Incubation with AGEs at the concentration of 200 μg/ml had no detrimental effect on cell viability and did not show any increase in ROS and proinflammatory cytokines such as IL-6 and TNF-α. In addition, AGEs appeared to have no direct stress effect on melanocytes when phosphorylated p38 expression was checked. However, AGEs did heighten melanin production, tyrosinase activity, and the expression of melanogenesis-associated molecules including MITF and tyrosinase. Additionally, sections of cultured human skin (by Fontana-Masson stain) showed proportionately higher melanin pigmentation by after AGEs exposure, based on ratios of pigmented epidermal areas to total epidermal areas (test samples vs. controls). The above results may indicate that AGEs could influence melanogenesis even at the concentration of AGEs, that does not affect ROS and proinflammatory cytokine production.

AGE-mediated signaling is initiated once the corresponding receptor for AGE (RAGE) is activated^44^. RAGE is expressed in various kinds of cells such as astrocytes, malignant glioma, endothelial cells, and fibroblasts^7^,^45^. To date, the details of RAGE expression in melanocytes have been unclear, but our study proved the existence of RAGE expression in melanocytes through western blot and immunofluorescence studies, suggesting its possible role in melanocytes. Melanogenesis through AGEs-RAGE pathway, which is confirmed by RAGE blockage experiments, is a new characterization of RAGE functions since RAGE is known to be involved in inflammation, cell migration, and ROS production. Thus, RAGE could be a crucial factor in melanogenesis through AGEs.

AGE-RAGE interaction is, in fact, associated with several signaling pathways, namely ERK1/2, phosphatidyl-inositol 3 kinase, Cdc42/Rac, and stress-activated protein kinase/c-Jun-NH2-terminal kinase (SAPK/JNK)^34^,^46^. Huttunen *et al*. have demonstrated that RAGE signaling also stimulates CREB phosphorylation (and ERK1/2 activation thereby) in separate neuroblastoma and embryonic stem cell lines^47^. On this basis, we explored the possibility that CREB phosphorylation, as a reputed feature of major MC1R-mediated melanogenic signaling^41^, might similarly be involved in melanocytic RAGE signaling. As p-CREB binds to the CRE consensus motif of MITF, expression levels of MITF and tyrosinase increase accordingly^48^,^49^,^50^. Following the exposure of melanocytes to AGEs, we detected increased phosphorylation of ERK (p-ERK) and CREB (p-CREB), which are downstream targets of RAGE signaling^51^. In addition, increased p-CREB expression and melanogenesis-associated signaling, including MITF expression, tyrosinase activity, and melanin production, in AGE-exposed cells were ablated through preincubation in anti-RAGE antibody, suggesting that CREB and pigmentation signaling due to AGEs are achieved via RAGE. Therefore, AGE-RAGE interaction may induce both ERK and CREB signaling, increase MITF expression and tyrosinase activity and ultimately boost melanin production in melanocytes.

In conclusion, our findings indicate that AGEs, secreted by keratinocytes in response to UV irradiation, are capable of stimulating melanogenesis through ERK and CREB signaling via RAGE. Moreover, RAGE plays a regulatory role in melanogenesis, rather than engaging in free radical production and proinflammatory cascades as previously asserted. Our discovery that AGE-RAGE interaction upregulates melanogensis through CREB-MITF-tyrosinase pathway in melanocytes illuminates a novel mechanism of melanogenesis. Although still in early stages of research, AGEs and RAGE signaling may be a promising target in preventing the melanogenesis accompanying photoaging

## Materials and Methods

### Cell cultures

Human foreskin samples were obtained after written informed consent and in accordance with a protocol approved by Yonsei University College of Medicine Institutional Review Board. PHEKs, PHEMs, and PHDFs were isolated from foreskin samples and maintained in keratinocyte basal medium (KBM) with all supplements (Lonza, Walkersville, MD, USA), melanocyte basal medium (MBM) with all supplements plus 200 nM 2-O-tetradecanoyl-phorbor-13-acetate (TPA; Sigma-Aldrich, St. Louis, MO, USA), and Dulbecco’s modified Eagle’s minimal essential medium (DMEM; Welgene, Daegu, Republic of Korea) supplemented with 10% fetal bovine serum (FBS; Welgene) and 1% penicillin-streptomycin (Welgene), respectively. For experiments, PHEKs, PHEMs, PHDFs were used at passage number between 3 and 5. Cells from 3 different donors with age of 10–13 were used in this study. The non-tumorigenic melan-a cell line of mouse melanocytes, gifted by Prof. Dorothy C. Bennett at St George’s Hospital Medical School, London, UK, was cultured in RPMI 1640 medium (Welgene), which contains 10% FBS, 1% penicillin-streptomycin, and 200 nM TPA. HaCaT cells, an immortalized human keratinocyte cell line, were cultured in DMEM supplemented with 10% FBS and 1% penicillin-streptomycin. All cells were maintained at 37 °C in a humidified 5% CO_2_ incubator.

### Reagents

AGE-BSA (Calbiochem, Darmstadt, Germany), which is a mix of Nε-(carboxymethyl)-lysine, pentosidine, and others, were used throughout this study. BSA was purchased from Sigma-Aldrich. For blocking RAGE, anti-RAGE antibody was from R&D Systems (Abingdon, UK).

### Western blot analysis

Whole cell extracts were lysed in RIPA buffer. Proteins were resolved by 10% SDS-polyacrylamide gel electrophoresis and transferred to nitrocellulose membranes, which were blocked by 5% skim milk in TBST and incubated with primary antibodies against MITF (Abcam, Cambridge, UK), tyrosinase (Santa Cruz Biotechnology, Dallas, TX, USA), p-CREB, CREB, p-ERK1/2, ERK1/2 (Cell Signaling Technology, Danvers, MA, USA), RAGE (Abcam), or glyceraldehyde 3-phosphate dehydrogenase (GAPDH; Santa Cruz Biotechnology) at 4 °C overnight. Appropriate secondary antibodies were added, and the protein bands were detected using an enhanced chemiluminescence reagent. Test blots were probed for test proteins and GAPDH in the same blot.

### Confocal microscopy

Melanocytes were washed in phosphate-buffered saline (PBS) and fixed in 4% paraformaldehyde (room temperature, 15 minutes). Samples were then washed in PBST (PBS + 0.1% Tween-20) three times and incubated overnight (4 °C) in anti-RAGE (R&D Systems) and anti-Melan-A (Leica Biosystems, Newcastle, UK). For human skin tissues, formalin fixed paraffin-embedded tissue sections were prepared for immunofluorescent staining. Skin tissues were incubated with anti-RAGE and Melan-A. All samples were mounted in a solution of 4′,6-diamidino-2-phenylindole (DAPI; Vector Laboratories, Inc., Burlingame, CA, USA). Slides were evaluated with confocal microscopy (LSM 700; Carl Zeiss, Oberkochen, Germany).

### Skin organ culture

Postsurgical remnants of human breast skin were obtained after written informed consent and in accordance with protocol approved by Yonsei University College of Medicine Institutional Review Board and prepared as previously described^52^,^53^. Samples were immersed in DMEM with 10% FBS and 1% penicillin/streptomycin. AGEs (200 μg/ml) were added twice (Day 1 and 3) during the 5-day incubation (37 °C, 5% CO_2_). Frozen tissue sections were then subjected to Fontana-Masson staining for melanin (Abcam kit). Melanin positive epidermal areas relative to total epidermal areas were measured by using Image J software (National Institutes of Health, Bethesda, MD, USA) based on previously reported protocols^54^,^55^. In brief, TIFF image files were opened in Image J and were adjusted to red, green and blue (RGB) color space in color threshold section. Total epidermal area was measured by analyze section in program. By defining minimum and maximum vales for each RGB color, positive areas of Fontana-Masson staining were displayed. Then, positive staining area was measured. We calculated the ratio of pigmented epidermal area (PA, Fontana Masson positive area)-to-total epidermal area (EA, total epidermal area).

### Melanin content

To assess the melanin production, melan-a cells and PHEMs were exposed to AGEs (200 μg/ml) or BSA for 4 days. The cell pellets were then incubated in 1 N NaOH (60 °C, 2 h). Absorbance at 405 nm (detected by ELISA reader) was compared with synthetic melanin standard curve (Sigma-Aldrich).

### Tyrosinase activity assay

Measurements of tyrosinase activity were performed in accordance with a previously reported method^56^. In brief, the cells were incubated (72 h) in AGEs (200 μg/ml) or BSA and lysed in radioimmunoprecipitation assay (RIPA) buffer. Tris-glycine sodium dodecyl sulfate (SDS) sample buffer without mercaptoethanol was added to lysed samples and then was resolved through 10% SDS-polyacrylamide gel electrophoresis. The gels were immersed in 0.1 M NaH_2_PO_4_ (pH 6.8, 30 min) and then buffer was replaced. After rinsing, a buffer with 5 mM L-DOPA (Sigma-Aldrich) was added to the gels and incubated (37 °C, 3 h) before visualizing the darkened electrophoretic bands.

### Real-time PCR

Total RNA was isolated from primary human epidermal melanocytes using RNeasy Plus Mini Kit (QIAGEN, Valencia, CA, USA). cDNA was synthesized using PrimeScript 1^st^ strand cDNA Synthesis Kit (Takara Bio, Otsu, Shiga, Japan) following the manufacturer’s protocol. Quantitative real-time PCR was performed using Power SYBR Green PCR Master Mix (Applied Biosystems, Foster City, CA, USA). The primers for PCR were as follows: RAGE 5′-GTG TCC TTC CCA ACG GCT C-3′ (forward) and 5′-ATT GCC TGG CAC CGG AAA A-3′ (reverse); GAPDH 5′-AAG GTG AAG GTC GGA GTC AAC-3′ (forward) and 5′-GGG GTC ATT GAT GGC AAC AAT A-3′ (reverse); IL-6 5′-TAG TCC TTC CTA CCC CAA TTT CC-3′ (forward) and 5′-TTG GTC CTT AGC CAC TCC TTC-3′ (reverse); TNF-α 5′-CTG AAC TTC GGG GTG ATC GG-3′ (forward) and 5′-GGC TTG TCA CTC GAA TTT TGA GA-3′ (reverse); β-actin 5′-GGC TGT ATT CCC CTC CAT CG-3′ (forward) and 5′-CCA GTT GGT AAC AAT GCC ATG T-3′ (reverse).

### Cytotoxicity assay

To check cell viability, the MTT 3-(4,5-dimethylthiazol-2-yl)-2,5-diphenyltetrazolium bromide assay (Sigma-Aldrich) was used. PHEMs and melan-a cells were incubated in differing concentrations of AGEs or BSA for 4 days. All cells in a 96-well plate were incubated with MTT stock solution (37 °C, 4 h). After labeling with MTT, the cells were incubated (10 min) in dimethyl sulfoxide (DMSO; Sigma-Aldrich), and absorbance of each sample was read at 570 nm.

### Cellular ROS measurement

To detect the production of ROS within the cells, flow cytometry was performed with LSRII (BD Biosciences, North Ryde, New South Wales, Australia). 20,70-dichlorofluorescein diacetate (H2-DCFDA; Molecular Probes, Eugene, OR, USA) was used. ROS production was compared between control, BSA and AGEs treated melan-a cells. H_2_O_2_ incubated cells were used as positive control. Data were analyzed using FlowJo (TreeStar, San Carlos, CA, USA).

### UVB irradiation

A Spectronics BLE-1T158 lamp (Spectronics Corp., Westbury, NY, USA) served as a UVB source. Keratinocytes were irradiated with 50 mJcm^−2^ UVB at room temperature.

### Statistical analysis

All values were expressed as means ± SD. Student’s t-test was used between two groups and ANOVA analysis was used for comparing multiple groups, with statistical significance set at *p* < 0.05. Standard software (SPSS; SPSS Inc., Chicago, IL, USA) was employed.

## Additional Information

**How to cite this article**: Lee, E. J. *et al*. Advanced glycation end products (AGEs) promote melanogenesis through receptor for AGEs. *Sci. Rep.* **6**, 27848; doi: 10.1038/srep27848 (2016).

## Supplementary Material

Supplementary Information
