# Supplementary material for: Advanced glycation end products (AGEs) promote melanogenesis through receptor for AGEs
Source: Sci Rep. 2016 Jun 13;6:27848. doi: 10.1038/srep27848 (PMC4904211; doi:10.1038/srep27848)
Supplement: Supplementary Information [file srep27848-s1.pdf]

Advanced glycation end products (AGEs) promote melanogenesis through receptor for AGEs

Eun Jung Lee, Ji Young Kim & Sang Ho Oh*

Department of Dermatology and Cutaneous Biology Research Institute, Severance Hospital, Yonsei University College of Medicine, Seoul, Korea

***Corresponding author:**

Sang Ho Oh

Department of Dermatology and Cutaneous Biology Research Institute, Severance Hospital, Yonsei University College of Medicine, Seoul, Korea

Tel: +82 2 2228 2080, Fax: +82 2 393 9157

E-mail: oddung93@yuhs.ac

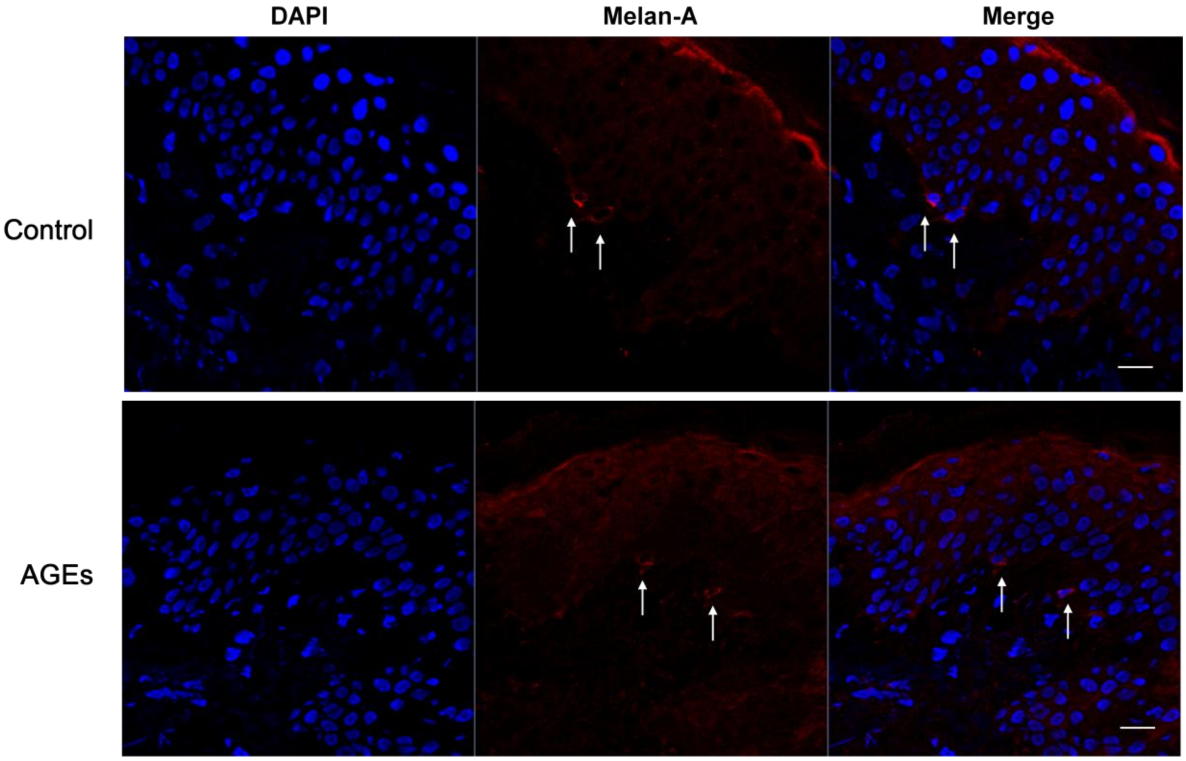

Figure S1. AGEs do not affect on number of melanocytes. Human skin cultures exposed to AGEs (200 μg/ml) twice (Day 1 and 3) in a 5-day period were stained with anti-Melan-A, representative marker for melanocytes. Arrows indicate Melan-A stained melanocytes. Scale bar represents 50 μm.

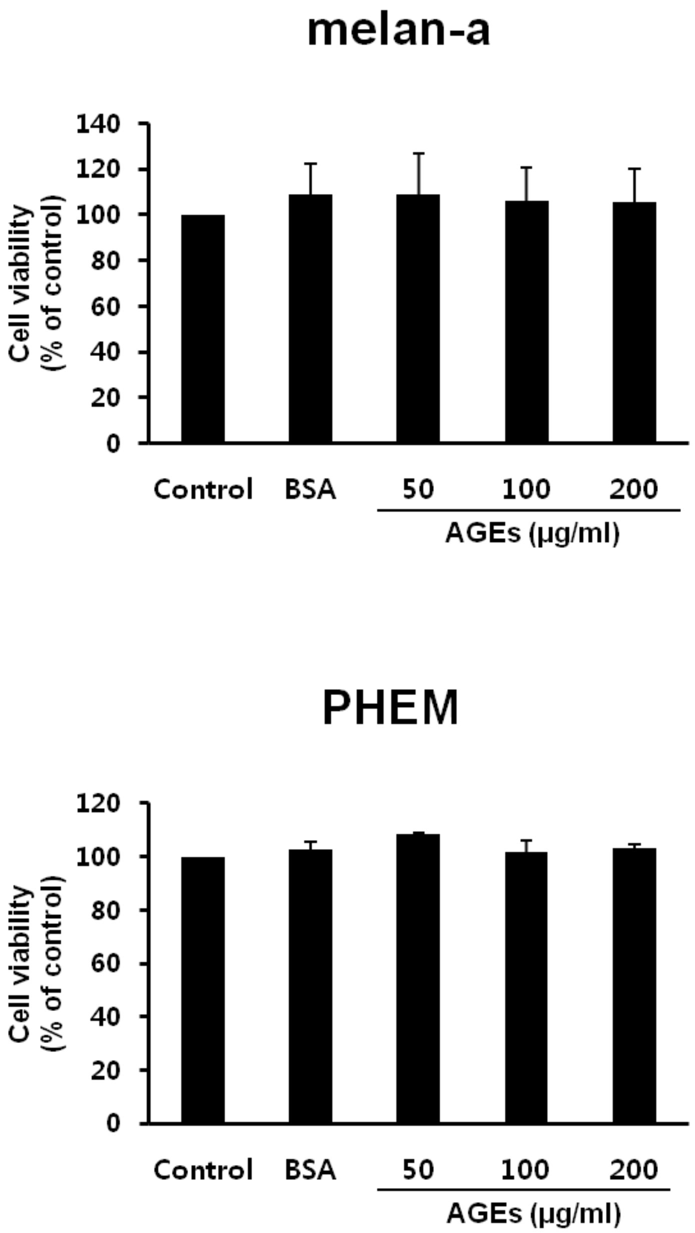

Figure S2. Effects of AGEs on cell viability in melanocytes. To evaluate the cytoxicity on melan-a cell line and PHEM, MTT test was performed. As shown in figure, AGEs treatment did not show toxicity. All data expressed as mean±SD of three independent experiments.

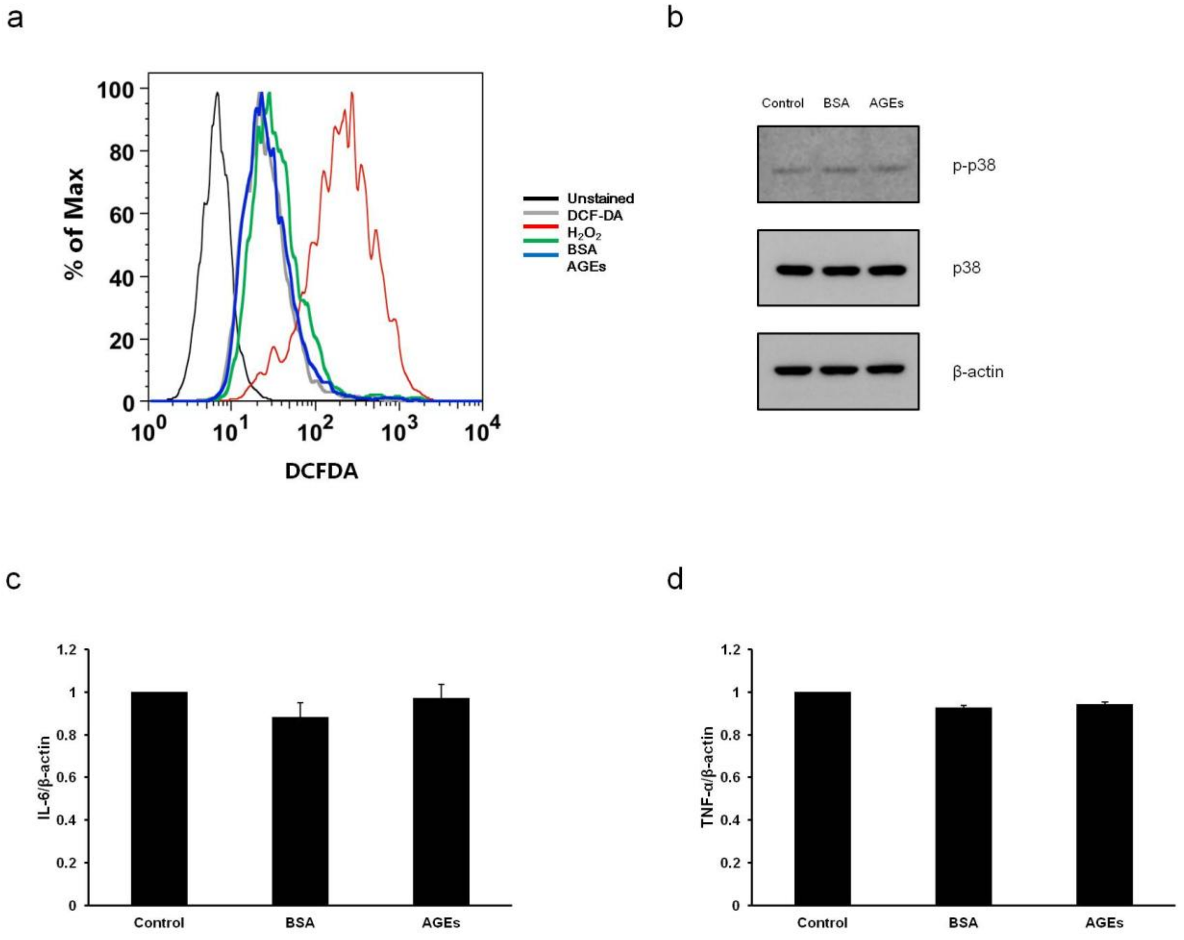

Figure S3. AGEs do not induce direct cellular stress in melanocytes. Melan-a cells were exposed to AGEs (200 μg/ml) for 24 h to measure (a) ROS level by FACS analysis. H_2O_2 treatment was used as positive control. (b) p38 response was confirmed after AGEs treatment for 15 min using western blot analysis. (c) IL-6 and (d) TNF-α levels in samples with AGEs incubation for 24 h were investigated by real-time PCR. All measurements reveal that AGEs do not trigger direct cellular stress to melanocytes.

melan-a

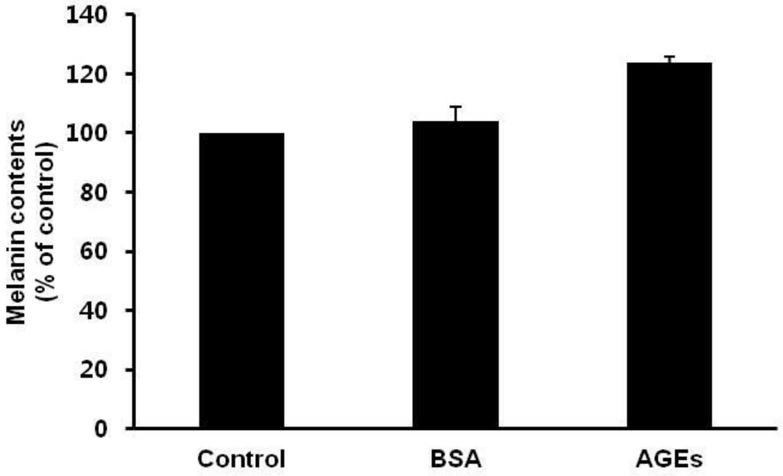

PHEM

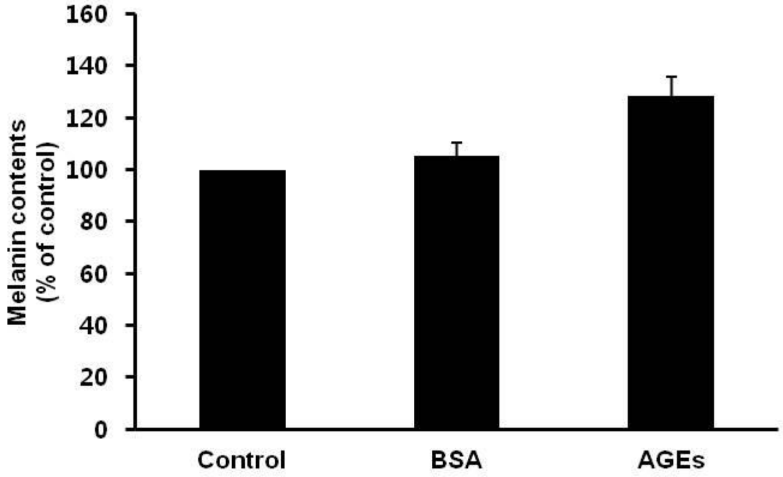

Figure S4. Effect of AGEs on melanin contents after two week treatment. AGEs (200 μg/ml) were treated to mouse and human melanocytes and melanin contents were measured after 2 week. AGEs-treated cells showed higher amounts of melanin compared to control.

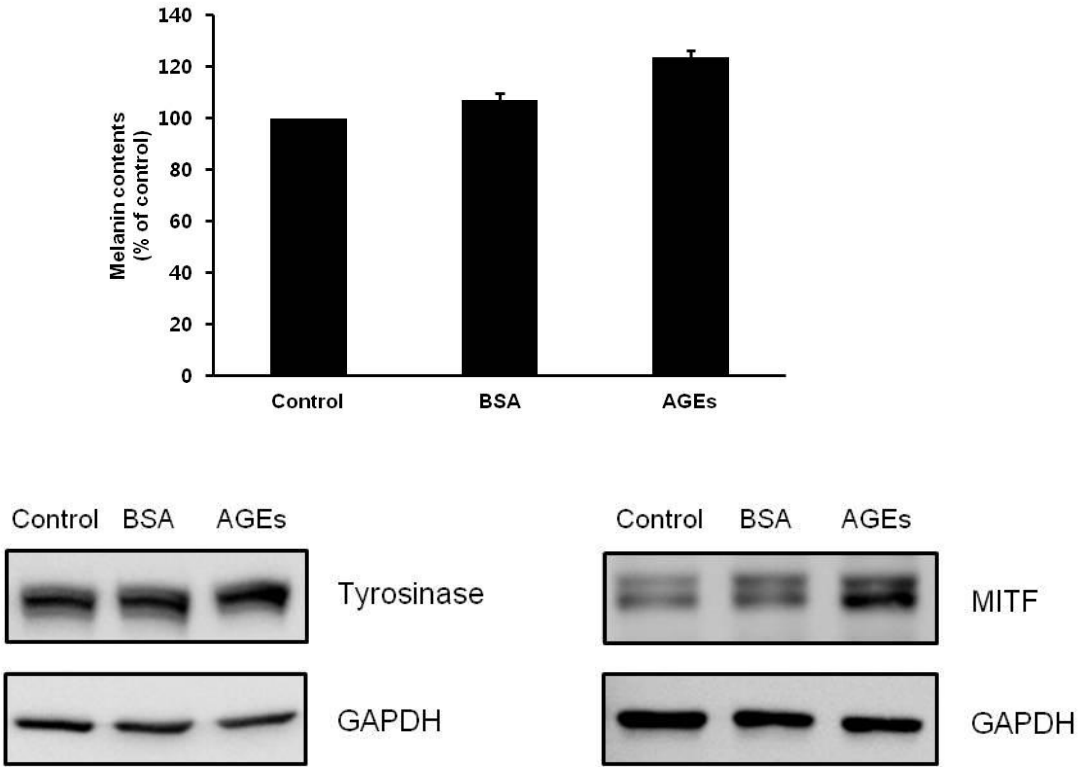

Figure S5 AGEs-induced melanogenesis in adult non-genital melanocytes. AGEs (200 μg/ml) were treated to melanocytes and melanin contents (72 h), tyrosinase (48 h) and MITF expression (4 h) were observed in same condition with foreskin melanocyte experiments.

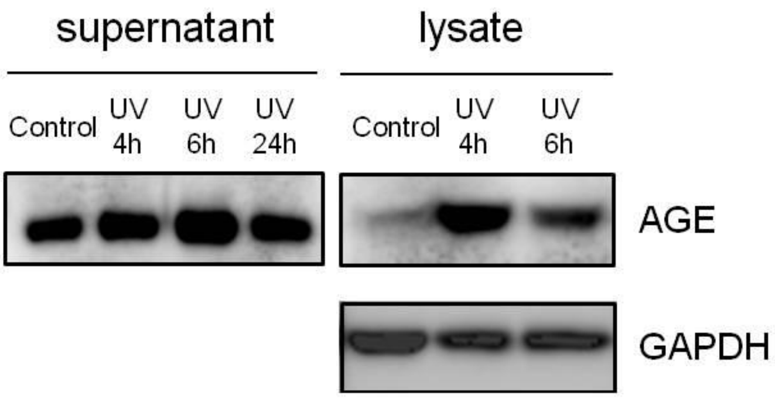

Figure S6. UVB radiation increases AGE production and secretion in HaCaT keratinocytes. To investigate whether AGEs are produced or secreted by keratinocytes in response to UVB radiation, 50mJcm^{-2} UVB was delivered to keratinocytes. Proteins from supernatant were precipitated by using trichloroacetic acid. After indicated incubation times, increased AGE expression was detected in supernatnats and lysates of keratinocytes exposed to UVB.
